# A Legume Genetic Framework Controls Infection of Nodules by Symbiotic and Endophytic Bacteria

**DOI:** 10.1371/journal.pgen.1005280

**Published:** 2015-06-04

**Authors:** Rafal Zgadzaj, Euan K. James, Simon Kelly, Yasuyuki Kawaharada, Nadieh de Jonge, Dorthe B. Jensen, Lene H. Madsen, Simona Radutoiu

**Affiliations:** 1 Department of Molecular Biology and Genetics, Faculty of Science and Technology, Aarhus University, Aarhus, Denmark; 2 Carbohydrate Recognition and Signalling (CARB) Centre, Aarhus, Denmark; 3 Ecological Sciences, The James Hutton Institute, Invergowrie, Dundee, United Kingdom; Virginia Tech, UNITED STATES

## Abstract

Legumes have an intrinsic capacity to accommodate both symbiotic and endophytic bacteria within root nodules. For the symbionts, a complex genetic mechanism that allows mutual recognition and plant infection has emerged from genetic studies under axenic conditions. In contrast, little is known about the mechanisms controlling the endophytic infection. Here we investigate the contribution of both the host and the symbiotic microbe to endophyte infection and development of mixed colonised nodules in *Lotus japonicus*. We found that infection threads initiated by *Mesorhizobium loti*, the natural symbiont of *Lotus*, can selectively guide endophytic bacteria towards nodule primordia, where competent strains multiply and colonise the nodule together with the nitrogen-fixing symbiotic partner. Further co-inoculation studies with the competent coloniser, *Rhizobium mesosinicum* strain KAW12, show that endophytic nodule infection depends on functional and efficient *M*. *loti*-driven Nod factor signalling. KAW12 exopolysaccharide (EPS) enabled endophyte nodule infection whilst compatible *M*. *loti* EPS restricted it. Analysis of plant mutants that control different stages of the symbiotic infection showed that both symbiont and endophyte accommodation within nodules is under host genetic control. This demonstrates that when legume plants are exposed to complex communities they selectively regulate access and accommodation of bacteria occupying this specialized environmental niche, the root nodule.

## Author Summary

Plants have evolved elaborated mechanisms to monitor microbial presence and to control their infection, therefore only particular microbes, so called “endophytes,” are able to colonise the internal tissues with minimal or no host damage. The legume root nodule is a unique environmental niche induced by symbiotic bacteria, but where multiple species, symbiotic and endophytic co-exist. Genetic studies of the binary interaction legume-symbiont led to the discovery of key components evolved in the two partners allowing mutual recognition and nodule infection. In contrast, there is limited knowledge about the endophytic nodule infection, the role of the legume host, or the symbiont in the process of nodule colonisation by endophytes. Here we focus on the early stages of nodule infection in order to identify which molecular signatures and genetic components favour/allow endophyte accommodation, and multiple species co-existence inside nodules. We found that colonisation of *Lotus japonicus* nodules by endophytic bacteria is a selective process, that endophyte nodule occupancy is host-controlled, and that exopolysaccharides are key bacterial features for chronic infection of nodules. Our strategy based on model legume genetics and co-inoculation can thus be used for identifying mechanisms operating behind host-microbes compatibility in environments where multiple species co-exist.

## Introduction

Plants are the major manufacturers of carbohydrates in ecosystems, and their roots develop in soil environments rich in heterotrophic microorganisms that require carbon for their growth. To adapt to this habitat, plants have evolved sophisticated surveillance systems for monitoring microbial presence, or invasion and corresponding response strategies [1–4]. As a consequence, only a limited range of microbes, endophytes and symbionts have the ability to colonise internal plant tissues with minimal or no host damage [5, 6].

The legume-symbiotic rhizobia interaction is a well-studied example of a very selective and clearly defined host/non-host plant-microbe association. Rhizobial-produced lipochitin oligosaccharide (Nod factors) are recognised by receptors in the host that subsequently trigger cell dedifferentiation, organogenesis and infection of root nodules [7–9]. In most legumes the infection starts at the stage of bacterial entrapment within curled root hairs. This is followed by initiation and elongation of infection threads (ITs), which are plant-derived tubular structures that guide the microbe through the plant’s epidermal and cortical cell layers towards the nodule primordia, in which the bacteria are endocytosed in organelle-like symbiosomes where they develop into bacteroids and fix nitrogen. Characterization of plant mutants impaired at different stages of the symbiotic process has identified genes required to establish and regulate this microbial infection. In *Lotus japonicus*, *SymRK*, *Nup133*, *Nup85*, *Nena*, *Castor* and *Pollux* act upstream of the nuclear calcium spiking induced by the symbionts and are required for nodule organogenesis [10–14]. A calcium-calmodulin dependent kinase, CCaMK, subsequently interprets these calcium oscillations and interacts with CYCLOPS to coordinate infection with organogenesis [15–17]. Activation of cytokinin signalling via the LHK1 receptor leads to cell division, and downstream transcriptional activators *Nsp1*, *Nsp2* and *Nin*, control both the infection and the organogenesis [18–21]. In *Lotus*, which has spherical determinate nodules with a transient meristem, genes involved in actin rearrangement or nucleation (*Nap1*, *Pir1*, *ArpC1*), a putative ubiquitin E3 ligase (*Cerberus*) and a pectate lyase (*Npl1*) are required for IT initiation and progression towards primordia, a process which also appears to be controlled by two genes, *Alb1* and *Crinkle*, whose products await identification [22–27]. Later in the developmental process several genes, for example *Sst1*, encoding a sulphate transporter, are required for bacterial persistence inside the plant cell [28], and *Medicago truncatula*, which develops indeterminate nodules with a persistent meristem, produces nodule-specific cysteine-rich (NCR) peptides to control the irreversible terminal differentiation of bacteria [29].

From the bacterial side, Nod factors are the main signals recognised by the host, but lipopolysaccharides (LPS), exopolysaccharides (EPS) and cyclic beta-glucans are also critical for infection and bacterial release inside the plant cells [30–32]. In addition, an array of species-specific bacterial effectors orchestrates another level of the specificity identified in the legume-rhizobia symbiosis [33–35].

Given that the final outcome of this highly controlled host-microbe interaction is the bacterial fixation of atmospheric nitrogen in exchange for plant-produced carbohydrates, it is surprising, that, as far as it is currently known, the host selects its symbiont on the basis of bacterial features that are not correlated with their capacity to fix nitrogen [36–39]. In accordance with this notion, inventories of bacterial species retrieved from nodules of legumes growing in a variety of environmental conditions and soils revealed a bacterial community composed of both symbionts and endophytes [40–46]. The presence of poor or even nonsymbionts within nodules of economically important legumes may thus negatively affect the efficiency of their symbiotic nitrogen fixation, and hence plant growth [47, 48]. However, to date there has been no evaluation of the role of endophytic bacteria in pioneer legumes grown in poor soils where fully compatible, highly efficient nitrogen fixing symbionts are either low in titre, or which might need to evolve into more effective symbionts [49]. Interestingly, recent experimental evolution studies have revealed that in the presence of the legume host as a selective environment, a more rapid evolution of symbiont compatibility takes place in a bacterial community [50]. Nevertheless, the co-habitation of diverse bacteria inside nodules raises questions with respect to endophyte recognition by the plant, their infection path(s), and the mechanisms employed by the host-symbiont-endophyte interacting partners leading to access and accommodation of endophytes. Currently there is limited information regarding the entry mode of endophytic bacteria and the role of the legume host, or the proficient symbiont, in the process of nodule colonisation by endophytes.

Here we report that in *Lotus* the colonisation of nodules by endophytic bacteria follows a selective process with at least three steps, that endophyte nodule occupancy is host-controlled, and that exopolysaccharides represent key bacterial features for chronic infection of nodules.

## Results

### *Lotus japonicus* nodules induced by *M*. *loti* can accommodate endophytes

**Fig 1:**
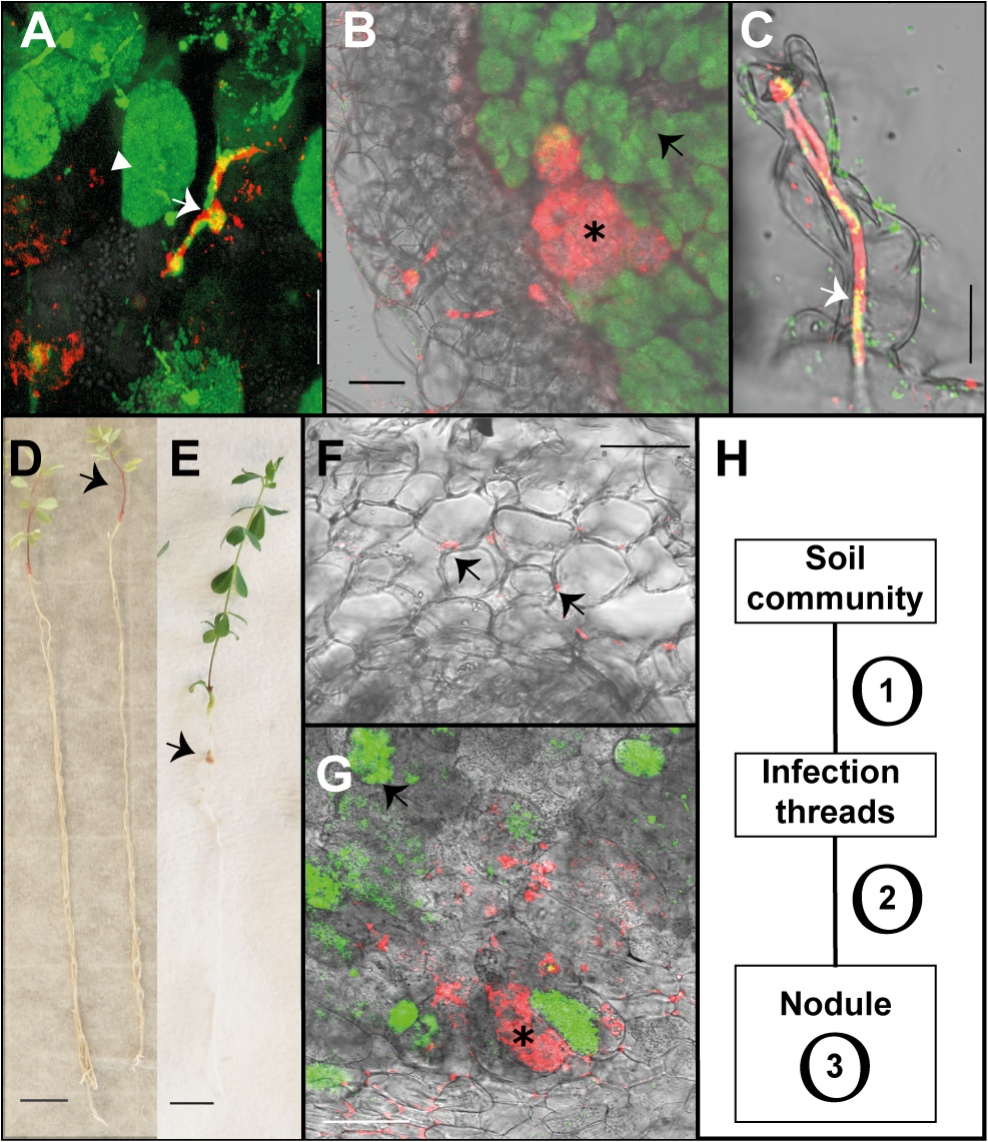
Endophytic colonisation of *Lotus japonicus* roots and nodules by *R*. *mesosinicum* KAW12. A) Nodule section displaying a cortical infection thread (arrow) that contains both the *M*. *loti* wild-type and KAW12 bacteria, among fully infected nodule cells (arrow head) containing the *M*.*loti* symbiont. B) Nodule section showing that KAW12 (*) multiplication inside nodules is limited to small sectors compared to *M*. *loti* wild-type (arrow), which is the predominant coloniser. C) Root hair infection thread (arrow) containing both the *M*. *loti* wild-type symbiont and the KAW12 endophytic bacteria. D) *Lotus* plants inoculated with KAW12 endophyte display a nod-minus and nitrogen-starved phenotype (arrow) in comparison to E) *Lotus* plants that form nodules (arrow) and establish a nitrogen-fixing symbiosis after inoculation with *M*. *loti* wild-type. F) Root section illustrating the capacity of KAW12 (arrow) to colonise the intercellular space of *Lotus* roots. G) Section of an *M*. *loti nodZ*-induced nodule presenting KAW12 (*) and *M*. *loti nodZ* (arrow) infection. H) The infection and accommodation of compatible endophytes within *Lotus* nodules is regulated in at least three steps. Scale bars: A) and C) 20 μm, B) F) and G) 50 μm, D) and E) 1 cm. *Mesorhizobium loti* bacteria are visualized in green, and KAW12 in red. Mixed inoculum has been used in A) to C) and G), and single inocula with the aforementioned bacteria have been used for E) to F).

In order to test the ability of endophytic bacteria to colonise and multiply inside *Lotus* nodules we chose to: i) investigate endophytic bacteria that were previously found inside plant roots, as endophytes or presumptive endophytes, and ii) monitor their ability to colonise nodules by visualising their presence inside primordia induced by the *M*. *loti* symbiont. In our tests we included *Herbaspirillum frisingense* GSF30, *Herbaspirillum* sp. B501 endophytic bacteria from *Miscanthus* and rice (*Oryza sativa*), respectively [51, 52], *Rhizobium giardinii* sp. 129E isolated from *Arabidopsis* roots [53], and *Burkholderia* sp. KAW25 (KAW25), *R*. *mesosinicum* KAW12 (KAW12) isolated from *Lotus* roots (see Material and Methods). None of these bacterial strains induced nodule formation when applied individually to *Lotus* roots. Fluorescently labelled endophytes and *M*. *loti* were mixed in a 1:1 inoculum, which was applied to *Lotus* seedlings. After nodule development, whole nodules, or hand sections were inspected microscopically for the presence of the two bacterial strains (Table 1). We found that, with the exception of *H*. *frisingense*, the other four strains were present inside the nodules or the cortical ITs induced by *M*. *loti* (Figs 1A and S1), but endophyte amplification and effective colonisation of the nodule interior was observed only for *Burkholderia* KAW25 and *Rhizobium* KAW12 (Figs 1B and S1C). These results show that in *Lotus*, the infection threads induced by *M*. *loti* can be inhabited by an endophyte (Fig 1A and 1C), and that particular bacteria have the capacity to employ this route for access into the nodules in which they multiply.

**Table 1:** Colonisation of *Lotus japonicus* nodules by endophytic bacteria.

Inoculum	Endophyte colonisation ratio colonised/total nodules (%)^a^	Symbiont colonisation ratio colonised/total nodules (%)	Frequency of plants with at least one nodule infected by the endophyte
*H. frisingense* GSF30 + *M. loti* wild type	0/32	n.d.	0/10
*Herbaspirillum* B501 + *M. loti* wild type	1/32^b^	n.d.	1/10 (10%)
*R. giardinii* 129E + *M. loti* wild type	3/1045 (0,29%)	n.d.	3/203 (1,5%)
*Burkholderia* KAW25 + *M. loti* wild type	8/245 (3,3%)	n.d.	7/88 (7,9%)
*R. mesosinicum* KAW12 + *M. loti* wild type	20/243 (8,2%)	n.d.	17/57 (29,8%)
*R. mesosinicum* KAW12 + *A. caulinodans*	4/231 (1,7%)***	8/231 (3,4%)	3/26 (11%)
*A. caulinodans* (n = 9)		2/153 (1,3%)	
*R. mesosinicum* KAW12 + *M. loti nodZ*	22/917 (2,4%)***	677/917 (74%)	19/153 (12%)
*M. loti nodZ* (n = 26)		203/252 (81%)	
*R. mesosinicum* KAW12 + *M. loti exoU*	1169/3588 (33%)***	510/3588 (14%)	195/199 (98%)
*M. loti exoU* (n = 171)		83/3653 (2,3%)	
*R. mesosinicum* KAW12 *eps1* + *M. loti* wild type	3/740 (0,4%)***	645/740 (87%)	3/100 (3%)
*R. mesosinicum* KAW12 *eps1* + *M. loti exoU*	0/2592 ***	75/2592 (2,9%)	0/139

n.d. not determined

a- statistical significance of *R*. *mesosinicum* inoculations compared to *R*. *mesosinicum* KAW12+*M*. *loti* wild type (***-P<0,005)

b- bacteria were found present inside this nodule in cortical infection threads

We observed that even when both symbiotic and endophytic bacteria were able to infect the nodule, the well-adapted symbiont, *M*. *loti*, occupied most of the nodule interior, while KAW12 or KAW25 remained within small, distinct sectors (Figs 1B and S1). Interestingly, the host response to the endophytic infection by KAW12 and KAW25 was different. Nodule sectors containing KAW25 bacteria were found to show signs of necrosis (S1C Fig), whilst no similar response was detected in the nodules containing KAW12 (Fig 1B). This indicates that infection and multiplication of endophytic bacteria within *Lotus* nodules is based on host-microbe compatibility.

Among the five different bacteria included in our study, KAW12 presented the highest level of nodule infection. One third of tested plants (29.8%) contained at least one KAW12-infected nodule (Table 1), and 20 out of the 243 analysed nodules (8.2%) were co-infected by KAW12, demonstrating the ability of this endophyte to colonise *Lotus* nodules.

These results based on analysis of a limited, but diverse set of endophytes show their differential capacity for nodule infection in the presence of *M*. *loti*, and that a sequential selection process shapes the community of bacterial inhabitants inside the nodules, i.e. i) access and/or persistence inside the IT, ii) within the nodule, iii) multiplication within the nodule without causing damage to the host.

### KAW12 is a nonsymbiotic *Rhizobium* with endophytic features

KAW12 was identified as a root-inhabiting bacterium in *Lotus* plants grown in Japanese forest soil (see Material and Methods), that causes no obvious effect (positive or negative) on its host (S3 Fig). A comparison of its 16S rRNA sequence against known bacteria revealed a close relationship to nodulating *Rhizobium* species (S2A Fig). In spite of this similarity to symbiotic bacteria, *Nod* and *Nif* gene clusters, including key symbiotic genes such as *nodC*, which is required for Nod-factor synthesis, and *nifH*, which encodes the Fe subunit of nitrogenase, were not found in the KAW12 genome (S2C Fig). In order to determine the type of infection that this bacterium, which neither produces Nod factors nor fixes atmospheric nitrogen, establishes with *Lotus*, the KAW12 derivative constitutively expressing DsRED was used for detailed analyses. Confirming the absence of symbiotic genes, KAW12 alone, or co-inoculated with *M*.*loti nodC* was unable to induce root hair curling or microcolony formation and was unable to nodulate *Lotus* (S3 Fig). A nitrogen-starved phenotype was observed when KAW12-inoculated plants were grown under low nitrogen conditions (1 mM KNO_3_) compared to plants inoculated with the effective symbiont *M*. *loti* (Fig 1D and 1E). However, careful inspection of KAW12-inoculated tissue revealed that KAW12 colonised the intercellular spaces of *Lotus* roots (Figs 1F and S3A). These results illustrate that KAW12 is a nonsymbiotic *Rhizobium* with endophytic features and a capacity for infecting symbiotic nodules.

### Endophytic invasion of nodules by KAW12 depends on a functional Nod factor-induced infection pathway

Research into the binary interaction between legumes and nitrogen fixing rhizobia has revealed that a number of molecular components produced by the bacteria are required and/or contribute to a successful symbiotic association. Nod factor, EPS, LPS, cyclic beta-glucans and Type-III Secretion System (T3SS) effectors have been shown to be major modulators of the host response [31]. The increased capacity of KAW12 to infect *Lotus* nodules, together with its apparent acceptance by the *Lotus* host, provided us with a unique opportunity to study the interplay between the legume host and various bacterial partners during mixed infections, and to identify molecular and genetic components contributing to nodule infection by endophytic bacteria.

In order to investigate if KAW12 has the capacity to launch an active infection once the symbiotic Nod factor signalling has been initiated in *Lotus* roots, we used two different symbiotic bacteria as co-inoculating partners i.e. *Azorhizobium caulinodans* ORS571, and a *M*. *loti nodZ* mutant. *Azorhizobium caulinodans* ORS571, a symbiont of *Sesbania rostrata* [54] induced root hair curling, microcolony formation, and a large number of nodule primordia (17 per plant), but approximately 99% of them remained uninfected (Table 1). Furthermore, ITs penetrating the primordia were not observed, indicating that the infection pathway induced by *A*. *caulinodans* Nod factors is only partly effective. This restricted symbiotic development was used as a background for assaying the contributions from KAW12 during infection. At 6 weeks after inoculation with *A*. *caulinodans* and KAW12 only 3 of 26 plants had nodules colonised by KAW12, and the overall frequency of colonisation was also very limited (4 out of 231 primordia) (Table 1 and S4 Fig). We then tested the ability of KAW12 to colonise the nodules induced by the *M*. *loti nodZ* mutant. This mutant strain produces Nod factors lacking the acetylated-fucosyl decoration, and as a consequence the induction of primordia and the infection process are delayed and less effective [55]. Inspection of plants inoculated with the *M*. *loti nodZ* and KAW12, showed that only 2.4% of the induced nodules were infected by the endophyte. This is more than 3-fold fewer than in the co-inoculation with the *M*. *loti* wild-type (8.2%). The frequency of plants containing at least one KAW12-colonised nodule was also reduced; 12% compared to 29.8% in the wild-type *M*. *loti* co-inoculation (Fig 1G and Table 1).

This lower frequency of bacterial infection in the absence of a fully functional Nod factor signalling indicates that signalling components possessed by KAW12 cannot complement nor bypass an ineffective Nod factor-dependent infection pathway.

### Exopolysaccharides are critical for symbiotic and endophytic nodule colonisation

**Fig 2:**
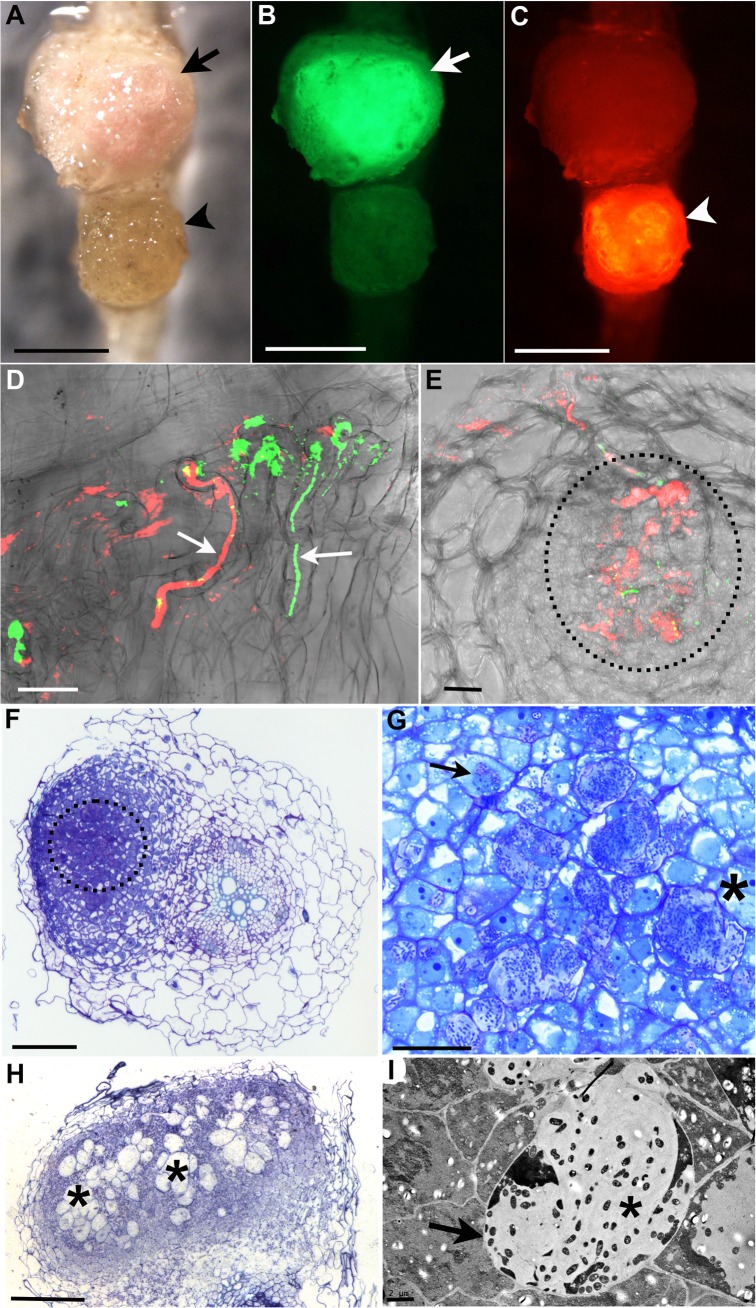
*R*. *mesosinicum* KAW12 colonisation pattern in *Lotus japonicus* nodules induced by *M*. *loti exoU*. A) to C) Nodules colonised by *M*. *loti exoU-*GFP (arrow) or KAW12-DsRed (arrowhead). The two nodules were visualized in bright field (A) with the GFP filter (B) or with the DsRed filter (C). D) Root hair infection threads (arrows) colonised by *M*. *loti exoU* (green) and KAW12 (red). E) Confocal laser scanning microscopy (CLSM) image of a nodule section illustrating internal nodule infection (dashed line) by KAW12 (red) and *M*. *loti exoU* (green). F) Thin section of a nodule primordium showing KAW12 infection (dashed line) in the inner zone. G) Detailed view of the same nodule as in (F) illustrating the inter- (*) and intra-cellular (arrow) KAW12-containing lagoons. H) Section of a mature nodule presenting multiple and enlarged lagoons colonised by KAW12 (*). (I) Transmission electron micrograph of an intercellular lagoon (arrow) containing bacteria surrounded by a white, undefined matrix (*). Scale bars = 500 μm (A to C), 20 μm (D, G), 50 μm (E), 100 μm (F, H), and 2 μm (I). The *M*. *loti exoU* is visualized in green and KAW12 in red (A to E).

In addition to Nod factor-induced signalling, host perception of compatible bacterial polysaccharides, such as EPS, is also important for symbiont recognition and efficient nodule infection [37, 56]. For example, in *Lotus*, perception of incompatible EPS produced by *M*. *loti R7A exoU* mutant severely impairs IT initiation and elongation is reduced, and consequently infected nodules are rare [32] (Table 1 and Fig 2A and 2B and S5A). Considering that Nod factor signalling is functional in the *Lotus*-*exoU* interaction [32], we investigated the ability of KAW12 to colonise nodules in the presence of incompatible symbiotic EPS signalling. Analysis of plants co-inoculated with *exoU* and KAW12 revealed that KAW12 had the ability to colonise the primordia and the ITs initiated by *exoU* bacteria (Table 1 and Fig 2A and 2C and Fig 2D). Infection threads colonised by KAW12 reached the base of the root hair where they expanded into an infection pocket, and from there, bacterial infection progressed into the underlying nodule primordium (Fig 2E). This indicates that the KAW12 endophyte has the capacity to rescue, and progress the arrested infection process induced by the *exoU*. Nodules infected by the *exoU*, KAW12, or by both bacteria, could be observed based on fluorescence marker screening (Fig 2A–2C). Unexpectedly, the majority of plants (98%) had at least one nodule containing KAW12, and overall 33% of primordia (1169 of 3588 nodules) were infected by KAW12, suggesting that molecular features of KAW12 may substitute for the lack of compatible *M*. *loti* EPS (Table 1). The increased frequency of KAW12-colonised nodules (33% compared to 8.2% in *M*. *loti* wild-type co-inoculation) also indicates that KAW12 infection is competitively restricted by the fully compatible EPS produced by wild-type *M*. *loti*.

The ability of KAW12 to overcome the arrested infection of the *exoU* suggested that KAW12 EPS might act as an important factor for its nodule colonisation ability. We tested this hypothesis by investigating the capacity of EPS-defective KAW12 to colonise the *exoU*-induced nodules. An EPS mutant of KAW12 was isolated from a random mutagenesis screen utilising the transposon mTn5-GNm [57]. The gene disrupted in this mutant encodes for a protein that shows high similarity (70%) to *PssN* from *R*. *leguminosarum* (S6A Fig), which is involved in polymerisation and export of EPS [58–60]. In contrast to the wild-type KAW12, the *eps* mutant displayed a non-mucoid colony growth phenotype, a typical characteristic of EPS deficiency (S6B Fig). *In planta* analyses of the colonisation phenotype revealed that this mutant, despite its presence inside root hair ITs when co-inoculated with the *exoU* (S6C Fig), was unable to infect and multiply within nodules, while *exoU* maintained its low infection ability (Table 1). In the reciprocal experiment, we found that co-inoculation of the KAW12 *eps* mutant with *M*. *loti* wild type enabled access of endophytes inside nodules, albeit to a very low frequency compared to EPS proficient KAW12 wild type bacteria (S6D Fig and Table 1). These results show that EPS is an important molecular feature of KAW12 allowing it to colonise the symbiont-induced primordium, and that co-infecting bacteria may complement each other for the lack of compatible EPS.

These co-inoculation studies pinpoint the critical role of EPS during nodule infection by symbiotic and endophytic bacteria, and have revealed that compatible EPS provides the wild-type symbiont with a clear advantage over the endophyte during mixed nodule infection.

### KAW12 is capable of intra- and inter-cellular colonisation of nodules

**Fig 3:**
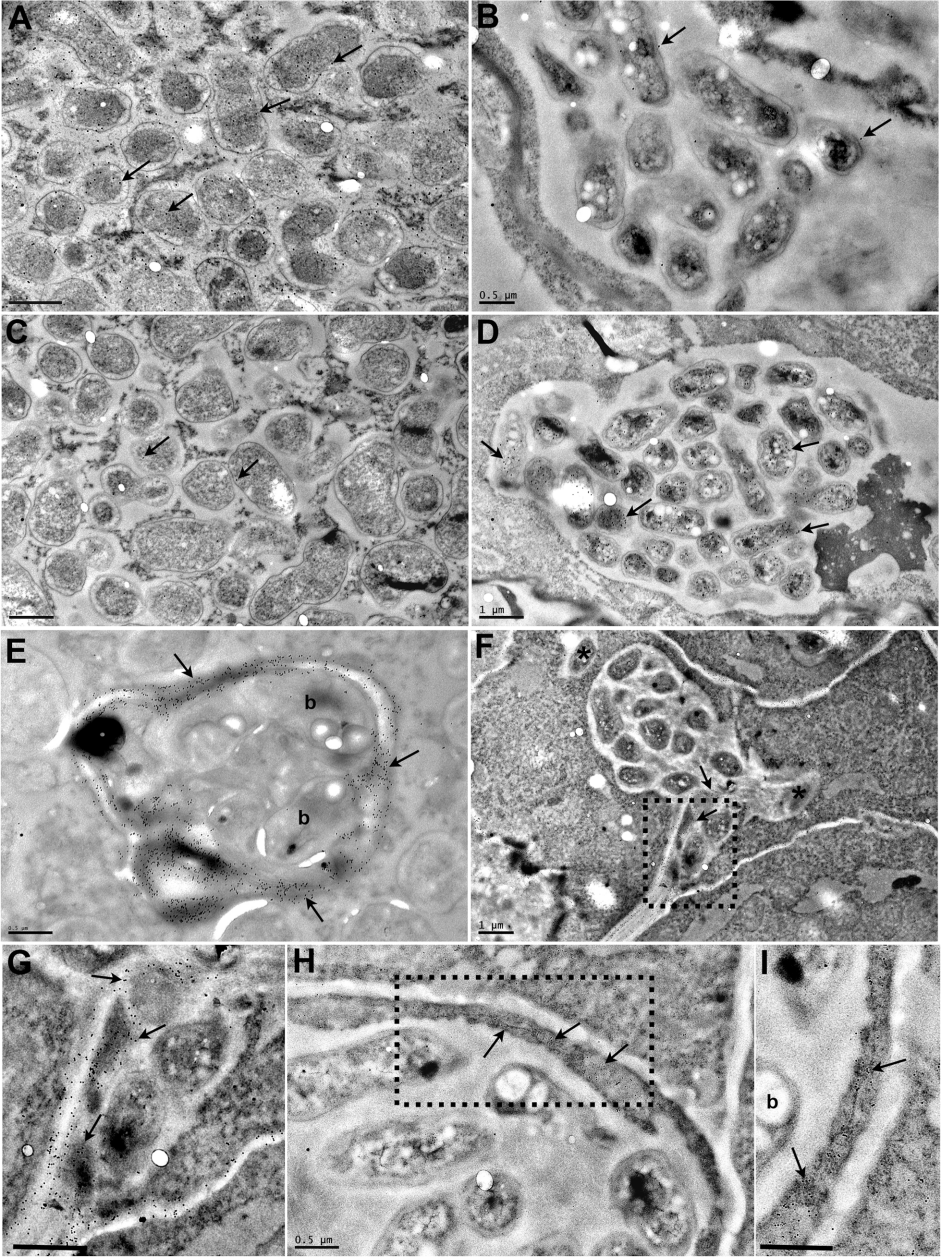
Transmission electron micrographs of *Lotus japonicus* nodules colonised by *M*. *loti exoU* or by *R*. *mesosinicum* KAW12. The nodule sections were immunogold labelled (arrows) with an antibody against the GFP protein (A, B), or against the DsRED protein (C, D). GFP was detected (arrows) in the *M*. *loti exoU*-selected nodule (A), and DsRED (arrows) in the KAW12-selected nodule (D). There is some minor nonspecific labelling by the GFP antibody (arrows) in the nodule colonised by the DsRed-tagged KAW12 (B) and by the DsRED antibody (arrows) in the nodule colonised by the GFP-tagged *M*. *loti exoU* (C). Immunogold labelling of homogalacturonan by the JIM5 monoclonal antibody shows the presence of cell wall material (arrow) in the infection thread that contains *M*. *loti exoU* (E), and in the lagoons containing KAW12 (F, G). KAW12 is released inside the plant cell (*) (F). Immunogold labelling of glycoproteins (arrows) by the MAC236 monoclonal antibody reveals their location within the plant cells containing the KAW12-containing lagoons (H, I). Detailed images of the regions marked by rectangles in F) and H) are shown in G) and I), respectively. Scale bars = 1 μm (A to D, and F), 0.5 μm (E, G, I). b = bacteria.

The nodule is a unique root organ where the intracellular accommodation and multiplication of compatible symbionts is permitted. Many of the nodules infected by KAW12 were abundantly colonised by endophytic bacteria in comparison to their sparse infection of the root intercellular spaces (Fig 1B and 1F). In spite of this increased nodule colonisation no signs of hypersensitive reactions or necrosis were observed in KAW12-colonised nodules (Fig 2A). This apparent acceptance of KAW12 endophytic bacteria by the host might be due to their distinct colonisation pattern within nodules that involves inter- and/or intracellular accommodation. To investigate this we studied the infection pattern of KAW12 in more detail using light and transmission electron microscopy (TEM) applied to selected nodules (Material and Methods). We observed that KAW12 multiplied extensively in the central zone of the nodules where bulbous structures accommodating numerous bacteria were observed between and within the plant cells (Figs 2F–2I and S5B). These disorganised structures differed in size and shape from the fully colonised nodule cells containing the *exoU* symbiont (Fig 3A and 3C) and from the finely defined ITs induced and occupied by symbiotic rhizobia (Figs 1C and 3B and 3D and S5C–S5H). Similar to the ITs induced by symbiotic bacteria, the KAW12-containing structures were encapsulated within cell wall material, as illustrated by the presence of a homogalacturonan epitope which is present in the plant cell wall and which was detected by the monoclonal antibody JIM5 (Figs 3E–3G and S5E and S5F). Glycoproteins, usually present in the IT matrix containing symbiotic bacteria and detected by the MAC236 antibody [61], were rarely observed in the matrix of KAW12-containing lagoons, but instead were found in the surrounding plant cells (Figs 3H and 3I and S5G and S5H). Localised cell wall degradation was observed leading to singular or multiple bacterial entrapments in the plant cell (Figs 3F and S5E–S5H). No membrane-like structure was observed around the internalised KAW12 indicating that symbiosomes were not formed. The infected plant cells contained KAW12 bacteria that were clustered together and surrounded by a white, undefined matrix (S5C–S5H Fig). The infected plant cells appeared to be viable, based on their apparently normal internal structure (S5F Fig), however, collapsed plant cells with massive intracellular infection of un-clustered KAW12 bacteria were also observed.

These results show that KAW12 is able to multiply within the nodules both intra- and inter-cellularly, and to a higher extent than that observed in the root tissue, indicating that nodules offer a competent biological niche for microbial accommodation.

### Plant symbiotic genes control invasion of nodules by endophytic bacteria

The results obtained from co-inoculation of *Lotus* wild-type plants showed that KAW12 has the ability to colonise ITs and nodules induced by *M*. *loti* and to progress the infection initiated by the *M*. *loti exoU* toward nodule primordia. In order to determine if plant genes required for infection by symbionts would also be necessary for the progression of the KAW12 infection, a panel of plant mutants impaired at different stages during symbiotic infection were analysed for their ability to sustain KAW12 colonisation. Mutation of a non-essential plant gene was assumed to result in a KAW12 infection frequency of nodule primordia similar to that of wild-type plants (i.e. 33%).

First, we analysed the *Cyclops*, *Cerberus*, *Nap1* and *ArpC1* genes involved in the signalling pathway controlling IT initiation and elongation. After the co-inoculation of mutants impaired in these *Lotus* genes by *exoU* and KAW12 only a negligible KAW12 infection of primordia was detected (Table 2), revealing that these plant symbiotic genes are essential for KAW12 infection of symbiotic nodules. These results confirm the dependency of KAW12 infection on the root hair IT initiation that is host-symbiont controlled. We then analysed the involvement of *Npl1*, *Alb1* and *Crinkle*, controlling symbiotic infection at the stage of IT passage through the epidermal/cortical barrier. After co-inoculation KAW12 was found impaired in infection of nodules induced by *exoU* on *npl1* and *alb1* mutants, but not on *crinkle* mutants where the infection frequency was similar to *Lotus* Gifu wild-type plants (Table 2). This indicates that the *Npl1* and *Alb1* genes, together with *Cyclops*, *Cerberus*, *Nap1* and *ArpC1* are required for both *M*. *loti* and KAW12 nodule infection via ITs. On the other hand, the mutation present in *crinkle*, which limits *M*. *loti* wild-type infection [23], does not affect KAW12 colonisation. This observation is interesting, since *alb1* and *crinkle* have been reported to have similar mutant phenotypes in the presence of wild-type *M*. *loti*, and, therefore, have been suggested to be impaired at corresponding stages of infection [26]. Identification of the *Alb1* and *Crinkle* genes would likely help to explain the observed differences. Finally, we investigated the role of the symbiotic gene *Sst1*, involved in the later stages of the *Lotus-M*. *loti* symbiosis. We observed that the *sst1* mutation had a limited effect on KAW12 colonisation, indicating that this gene is not required for KAW12 multiplication inside nodules (Table 2).

**Table 2:** Colonisation of *M*. *loti exoU* induced nodules by *R*. *mesosinicum* KAW12 on *Lotus japonicus* wild type and symbiotic mutants.

Genotype	Inoculum	Nr. of analysed plants	KAW12 colonisation ratio colonised/total nodules (%) ^a^	*M*. *loti exoU* colonisation ratio colonised/total nodules (%)	Frequency of plants with at least one nodule infected by KAW12
Wild-type ^b^	KAW12+*M*. *loti exoU*	199	1169/3588 (33%)	510/3588 (14%)	195/199 (98%)
	*M*. *loti exoU*	171		87/3653 (2.4%)	
*cyclops*	KAW12+ *M*. *loti exoU*	88	4/1423 (0.3%)***	4/1423 (0.3%)	3/88 (3.4%)
	*M*. *loti exoU*	74		0/1462	
*nap1*	KAW12+ *M*. *loti exoU*	57	1/724 (0.1%)***	0/724	1/57 (1.8%)
	*M*. *loti exoU*	38		0/406	
*arpC1*	KAW12+ *M*. *loti exoU*	65	5/748 (0.7%)***	0/748	5/65 (7.7%)
	*M*. *loti exoU*	12		0/162	
*cerberus1*	KAW12+ *M*. *loti exoU*	89	2/1327 (0.2%)***	0/1327	1/89 (1.1%)
	*M*. *loti exoU*	63		0/1292	
*npl1-1*	KAW12+ *M*. *loti exoU*	96	7/2047 (0.3%)***	0/2047	5/96 (5.2%)
	*M*. *loti exoU*	57		0/1175	
*alb1*	KAW12+ *M*. *loti exoU*	66	4/1460 (0.3%)***	0/1460	4/66 (6%)
	*M*. *loti exoU*	54		7/1180 (0.6%)	
*crinkle*	KAW12+ *M*. *loti exoU*	78	421/1446 (29%)	221/1446 (15%)	74/78 (95%)
	*M*. *loti exoU*	60		40/1215 (3.3%)	
*sst1*	KAW12+ *M*. *loti exoU*	69	509/1202 (42%)*	186/1202 (15%)	63/69 (91%)
	*M*. *loti exoU*	45		86/1112 (8%)	

a- statistical significance compared to wild-type inoculated with *R*. *mesosinicum* KAW12+ *M*.*loti exoU* (*P<0.05; ***P<0.005)

b-values for wild-type Gifu from Table 1 are presented for comparison with the symbiotic mutants.

Taken together, our analyses revealed that the legume host controls the access into nodules for both symbionts and endophytes when ITs are used as entry route, and that selective mechanisms may exist to control the accommodation of compatible symbionts and/or endophytes.

## Discussion

Land plants develop their root systems in a microbe-rich soil environment and have sophisticated mechanisms for microbial surveillance. In addition to the selection pressure imposed from the plant host, differences in the physiology of microbes and their ability to establish various microbe-microbe interactions, contribute to the composition of microbial communities in the soil, rhizosphere and *in planta* [4, 62–65]. There is a large diversity and wealth of diazotrophs in the soil, but it has become clear in the last decades that legumes select the infecting root nodule symbionts on the basis of molecular signatures, such as Nod factors, EPS, and LPS, that are unrelated to their symbiotic function performed within nodules [31]. As a consequence of this indirect selection mechanism, legumes that grow in natural habitats end up hosting a varied bacterial community inside nodules [45]. Experimental data support this suggestion; efficient nitrogen fixing bacteria, but also poor nitrogen fixers and endophytes have been shown to co-exist as part of the nodule bacterial community [42]. Likewise, laboratory studies using defined mixed symbiotic inocula and field studies monitoring the symbionts within nodules have revealed that mutants or poor nitrogen-fixing symbionts can infect and colonise nodules together with compatible strains [32, 66, 67]. Previous reports presented theoretical models or experimental evidence for the various mechanisms employed by the host to sanction the non/poor symbionts after establishment within nodules [68–70]. Our study focuses on the early stages of nodule infection by the endophytes in order to identify which molecular signatures and genetic components favour/allow an endophytic nodule infection.

Using co-inoculation experiments with a panel of endophytic bacteria together with the efficient symbiont *M*. *loti*, we show that complex host-microbe and microbe-microbe interactions can be captured and studied in *Lotus* plants grown under controlled conditions. Additional information may be gained from similar studies in legumes where rhizobial infection doesn’t follow the well-characterised root hair infection pathway. Using fluorescently labelled bacteria we monitored microbial infection patterns, and found that in the presence of *M*. *loti* the infection and accommodation of compatible endophytes within *Lotus* nodules is regulated in at least three steps (Fig 1H). Four of the tested endophytes were able to colonise cortical ITs induced by *M*. *loti* while only two infected and multiplied inside the nodules. Finally, *R*. *mesosinicum* KAW12 persisted inside nodules without inducing necrosis. Since KAW12 lacks the crucial genetic basis for establishing a nitrogen-fixing symbiosis a tempting explanation for this competence could reside in the endophytic features that enable KAW12 to colonise the intercellular spaces of *Lotus* roots in the absence of *M*. *loti*. Rhizobial species are frequently found as endophytes in a wide range of plant species [53, 71–75], indicating either an improved fitness compared to other soil bacteria or a better communication with the plant host. Nevertheless, the fact that the two *Rhizobium* species included in our study differ in their ability to colonise the nodules demonstrates that specific bacterial determinants contribute to their acceptance by the host.

Co-inoculation studies revealed that even if the endophyte had the competence to infect and multiply within nodules it was the *M*. *loti* symbiont which occupied most of the nodule interior, demonstrating its adapted ability to compete with other bacteria and to efficiently communicate with the host during infection (Fig 1B). The ability of KAW12 to co-infect the nodules in the presence of *M*. *loti* provided the opportunity to study the role/contribution of the symbiont and the host to endophyte infection.

*Rhizobium* KAW12 utilises *M*. *loti*-induced ITs as a route for access into the nodules (Fig 1C) and this infection pattern prompted us to investigate the role of the Nod factor signalling induced by the *M*. *loti* symbiont for the endophyte infection. Symbiotic rhizobia produce Nod factors continuously during root and nodule infection, and previous studies have revealed that fully compatible Nod factor signalling is important for the initiation and fast progression of ITs towards nodule primordia to ensure rapid infection and symbiotic development [54, 76, 77]. Our results from the co-inoculation experiments of *Lotus* with KAW12 and symbionts, such as *A*. *caulinodans* ORS571 or the *M*. *loti nodZ* mutant that produces less-compatible Nod factors, revealed a lower rate of KAW12 infection when compared to its co-inoculation with wild-type *M*. *loti* R7A. Based on these results we conclude that fully compatible Nod factor signalling is important for nodule infection by KAW12, as it allows rapid access of the endophyte into the nodule primordium.

We then investigated the role of bacterial exopolysaccharides, and demonstrated that during nodule infection compatible EPS provides the symbiotic bacteria with an advantage over the co-infecting endophyte. Both the frequency and the nodule volume presenting endophytic infection increased after co-inoculation with KAW12 and the *M*. *loti exoU* (Table 1 and Fig 2). Furthermore, KAW12 had the ability to rescue the *exoU*-containing aborted ITs within the root hairs and thus to progress the infection towards the nodule primordia in a manner similar to a *nodA* mutant of *M*. *loti* defective for Nod factor production [32]. Using a KAW12 *eps* mutant we show that this ability to bypass the requirement for compatible symbiotic EPS is dependent on KAW12 EPS. This is consistent with results showing EPS to be crucial for legume colonisation by symbiotic nitrogen-fixing bacteria, where both a protective activity against host defence responses and a positive signalling role have been proposed [32, 37, 56]. Interestingly, genes involved in EPS biosynthesis or export were found as targets of selection among several *Sinorhizobium medicae* and *S*. *meliloti* strains that share host plants [78]. Our results demonstrate that EPS represent a key molecular feature during nodule infection by both symbiotic and endophytic bacteria, and opens up the possibility that nodule infection by KAW12 is facilitated by perceptions of endophytic, yet compatible, EPS by the host.

Additional indications for a compatible host-endophyte interaction came from the analyses aimed to determine the role of the legume host for KAW12 infection. We found that the ability of KAW12 to progress the *exoU*-arrested ITs and to infect nodule primordia is dependent on early symbiotic genes. *Lotus* mutants impaired in the early events required for IT initiation and elongation (*Cyclops*, *Cerberus*, *Nap1*, *ArpC1*, *Npl1*, *Alb1*) were also defective for the endophyte infection (Table 2). We conclude that these host symbiotic genes gate the access of both symbiotic and endophytic microbes. On the other hand, genes like *Crinkle* that are required for *M*. *loti* infection, and *Sst1* which supports the symbiotic function of *M*. *loti* within nodules, did not seem to be required for KAW12 colonisation, indicating specific mechanisms operating inside host nodules to control the persistence of both symbiotic and endophytic bacteria.

Maintaining populations of highly competitive symbionts in the soil, and ensuring predominant occupancy by effective nitrogen fixing bacteria represents a major challenge that limits legume cultivation [79–82]. So far, most progress was obtained from the application of selected bioinoculants that are edaphically adapted [82, 83]. However, conventional application of superior nitrogen-fixing rhizobia did not prove to be a consistent solution for this challenge [84–86]. On the other hand, when the legume-breeding programme was performed in the presence of elite-selected rhizobia, and in conditions that favoured biological nitrogen fixation, a significant and consistent increase in plant yields was obtained as a result of selection for improved host-symbiont compatibility [87]. These practical results, together with those provided by the biodiversity studies corroborate with the results we present here and provide an explanation i.e.; the legume nodule is a unique environmental niche with an adapted program for accommodation of host-selected compatible soil microbes, and layers of compatibility determine access and colonisation efficiencies, symbiotic or not.

Our study shows that genetic resources available for the model legumes, in combination with co-inoculation strategies provide a reliable framework for identifying the genetic mechanisms operating behind this compatibility at the plant root interface, thus allowing developments to further address this challenge in a targeted manner.

## Material and Methods

A detailed version of material and methods is presented in the S1 Text.

### Plant material and bacterial strains

Plant genotypes and bacterial strains used in this work are listed in S1 Table and S2 Table.

### Isolation of *R*. *mesosinicum* KAW12 and *Burkholderia* sp. KAW25

Forest soil (0-4cm) was sampled from the Botanical Garden of Tohoku University (12–2 Kawauchi Aoba-ku Sendai Miyagi, Japan) on December 2006. Sterilized seeds of the *L*. *japonicus cCaMK* (*Ljsym72*) mutant were incubated with the soil in Magenta containers for 3 months. Plants were grown in 16h/light and 8h/dark conditions at 25°C. Whole plants were surface sterilized using 0.5% bleach and homogenized with 10 ml sterilized water. 500μl of the homogenated samples were inoculated onto sterilized seeds of the same mutant in Magenta containers with sterilized vermiculite supplemented with B&D medium, and were incubated for two months. Whole plants were sterilized and homogenized. These homogenized samples were plated onto TY medium, and KAW12 together with KAW25 were isolated among the bacteria growing on the plates. The 16S rRNA from KAW12 and KAW25 has been PCR-amplified, sequenced and analysed for similarity to other bacterial sequences present in the NCBI database (S2A and S2B Fig). According to the results of the 16SrRNA-gene sequences, the *Burkholderia* sp. KAW25 belongs to the plant-associated branch of the genus *Burkholderia*, [88] while the *Rhizobium* sp. KAW12 is within the *Rhizobium mesosinicum* species.

### Isolation of *R*. *mesosinicum* KAW12 *eps1* mutant

The KAW12 *eps1* mutant was isolated from a random mutagenesis screen utilising the transposon mTn5-GNm [57]. The transposon insertions site was identified by arbitrary PCR and sequencing. The KAW12 *eps1* was found to harbour an insertion in a gene encoding a polysaccharide export protein with 70% amino acid identity to PssN of *R*. *leguminosarum* bv. *trifolii* (S6A Fig). The mutant strain distinguished from wild-type KAW12 by displaying nonmucoid colony growth on YMB and G/RDM media.

### Plant assays and analyses

Plants were grown under nitrogen-limited conditions (1mM KNO_3_) and analysed for infection after 5 to 6 weeks. For the co-inoculation experiments a 1:1 mixture of bacteria (OD_600_- 0.02) was used. Screening for colonised nodules was performed by whole plant inspection on a Leica M165FC stereomicroscope in bright field and using filters for GFP and DsRED. Selected nodules were fine-sectioned (50 μm) using a Leica VT1000S vibratome, and analysed for internal colonisation with a Zeiss LSM510 Meta microscope. Semithin nodule sections were analysed by light microscopy.

### Transmission electron microscopy (TEM)

Ultrathin nodule sections were analysed by transmission electron microscopy (TEM) as previously described (Madsen et al., 2010). Commercially available DsRED and GFP antibodies were used to identify the bacteria on TEM sections (Fig 3A to 3D) via immunogold labelling [89].

## Supporting Information

S1 Fig Nonsymbiotic infection of *M*. *loti* induced nodules. (A) Confocal image of nodule section showing the presence of *Herbaspirillum* B501 strain (in green) inside IT (arrow) formed and colonized by *M*. *loti* (in red). Scale bar: 20 μm. (B) Confocal image of nodule section showing the presence of *Burkholderia* KAW25 strain (in green) inside IT (arrow) and within nodules induced and colonized by M. loti (in red). Scale bar: 20 μm. (C) Lotus nodule displaying signs of necrosis when colonized by KAW25. Left. Image of the whole nodule in bright field with necrotic sign (asterisk). Scale bar: 500 μm. Right. Nodule section visualized in bright field (top), with DsRed filter (middle), or GFP filter (bottom) shows the presence of *M*. *loti* (in red) and KAW25 (in green). Scale bars: 500 μm. D) Lotus nodule co-infected by *M*. *loti* and *R*. *giardinii* 129E visualized in bright field (top), with DsRed filter (middle), or GFP filter (bottom) shows the presence of both the nonsymbiont (in red) and the symbiont (in green). Scale bars: 500 μm.
(TIFF)

S2 Fig KAW12 and KAW25 are closely related to nodulating species, but lack key genes required for symbiosis. A) Phylogenetic relationship of KAW12 to other rhizobia strains based on 16S rRNA sequence. Bootstrap values are displayed on the tree nodes. (B) Phylogenetic relationship of KAW25 to other *Burkholderia* strains based on 16S rRNA sequence. Bootstrap values are displayed on the tree nodes. (C) Southern blot analysis illustrating the presence of *NodC* (left) and *NifH* (right) genes in the *M*.*loti*, but not in KAW12 and KAW25 bacteria. Bacterial DNA was digested with *HindIII*, *BamHI* or *EcoRI* restriction enzymes.
(TIF)

S3 Fig KAW12 colonisation of *Lotus* roots and nodules. (A) KAW12 colonises *Lotus* roots endophytically. The arrow marks the presence of bacteria labelled with DsRED inside the root visualised with a fluorescent microscope (scale bar 500 μm). (B) Confocal image of a nodule section showing that KAW12 maintains its nodule colonisation capacity when is labelled with the GFP fluorescent protein (asterisk) and the nodule-inducing *M*. *loti* wild-type symbiont is labelled with the DsRED (arrow) (scale bar 50 μm). (C) KAW12DsRED isolated by antibiotic selection from infected nodules similar to the one presented in Fig 1B) induces a Nod minus, nitrogen starved phenotype (compare to Fig 1E) when applied to new *Lotus* plants (scale bar 1cm). (D) The isolated bacteria display DsRED fluorescence (arrow) when roots from (C) are visualised on fluorescence microscope (scale bar 200 μm). (E) KAW12 alone, or coinoculated with *M*.*loti nodC* (n = 45) was unable to induce root hair curling or microcolony formation (scale bar 100 μm).
(TIF)

S4 Fig KAW12 colonisation of the nodules induced by *A*. *caulinodans* ORS571. Whole nodule primordia visualised in bright field (A), with a GFP filter (B) or with a DsRED filter (C) showing the presence of *A*. *caulinodans* (arrow in B) and KAW12 (arrow in C) inside the primordia. Section of a nodule primordia induced by *A*. *caulinodans* and colonised by *A*. *caulinodans* (arrow in D) and KAW12 (arrow in E). *A*. *caulinodanus* GFP is visualised in green and KAW12DsRED in red. Scale bars in A to C- 500 μm.
(TIF)

S5 Fig KAW12 and *M*. *loti exoU* colonisation patterns inside nodules induced by *M*. *loti exoU*. (A) Thin section of a mature nodule induced and colonised by *M*. *loti exoU*GFP displaying the infected cells (*) in the central zone. (B) Thin section of two closely developed nodule primordia (dashed lines) induced by *M*. *loti exoU* colonised by KAW12 (*). Compare with Fig 2H) to observe endophytic colonisation developing from the inner zone of the nodule. (C) and (D) Transmission electron micrographs of *M*. *loti exoU* induced nodules colonised by KAW12DsRED showing the immunogold labelling of KAW12 (arrows) using a DsRED antibody. (E) and (F). Transmission electron micrographs of *M*. *loti exoU* induced nodules colonised by KAW12DsRED showing the homogalacturonan epitope detection (arrows) using JIM5 antibody. (G) and (H). Transmission electron micrographs of *M*. *loti exoU* induced nodules colonised by KAW12DsRED showing the glycoprotein detection (arrows) using MAC236 antibody. Notice that KAW12 bacteria are surrounded by white undefined matrix (m) (C to H). Insets in (E) to (H) highlight the regions of interest.
(TIF)

S6 Fig KAW12 mutation in *eps1* leads to defective nodule colonisation phenotype. (A) Aminoacid alignment of KAW12 EPS1 predicted protein and the PssN protein from *R*. *leguminosarum* bv. *trifolii*. (B) The KAW12-*eps1* mutant displays a non-mucoid phenotype when grown on plates. (C) The DsRED labelled KAW12-*eps1* mutant colonises the root hair ITs (arrow) induced by *M*.*loti exoU* (scale bar 20μm). (D) The DsRED labelled KAW12-*eps1* mutant colonises the root hair IT (arrow) and, with very low frequency, the nodules induced by *M*.*loti* wild type R7A_GFP. Nodule cell walls are visualised in blue using the DAPI filter on the confocal microscope (scale bar 50μm).
(TIF)

S1 Text Supporting material and methods. (DOCX)

S1 Table Plant genotypes and primers used for genotyping *Lotus* mutants. (DOCX)

S2 Table Bacterial strains used in this study (A) and primers used for specific bacterial DNA amplification (B). (DOCX)

S3 Table Real time PCR CP values for 16S rDNA and *NodC* on nodule primordia selected based on microscopy fluorescence. (DOCX)
