# Supplementary figures and images for: A Legume Genetic Framework Controls Infection of Nodules by Symbiotic and Endophytic Bacteria
Source: PLoS Genet. 2015 Jun 4;11(6):e1005280. doi: 10.1371/journal.pgen.1005280 (PMC4456278; doi:10.1371/journal.pgen.1005280)

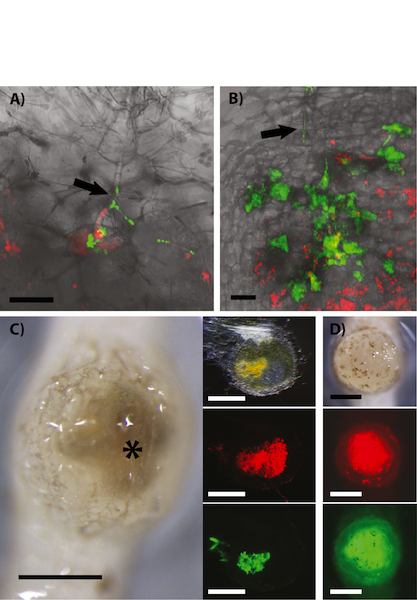

Supplement: S1 Fig — (A) Confocal image of nodule section showing the presence of Herbaspirillum B501 strain (in green) inside IT (arrow) formed and colonized by M. loti (in red). Scale bar: 20 μm. (B) Confocal image of nodule section showing the presence of Burkholderia KAW25 strain (in green) inside IT (arrow) and within nodules induced and colonized by M. loti (in red). Scale bar: 20 μm. (C) Lotus nodule displaying signs of necrosis when colonized by KAW25. Left. Image of the whole nodule in bright field with necrotic sign (asterisk). Scale bar: 500 μm. Right. Nodule section visualized in bright field (top), with DsRed filter (middle), or GFP filter (bottom) shows the presence of M. loti (in red) and KAW25 (in green). Scale bars: 500 μm. D) Lotus nodule co-infected by M. loti and R. giardinii 129E visualized in bright field (top), with DsRed filter (middle), or GFP filter (bottom) shows the presence of both the nonsymbiont (in red) and the symbiont (in green). Scale bars: 500 μm. (TIFF) [file pgen.1005280.s001.tiff]

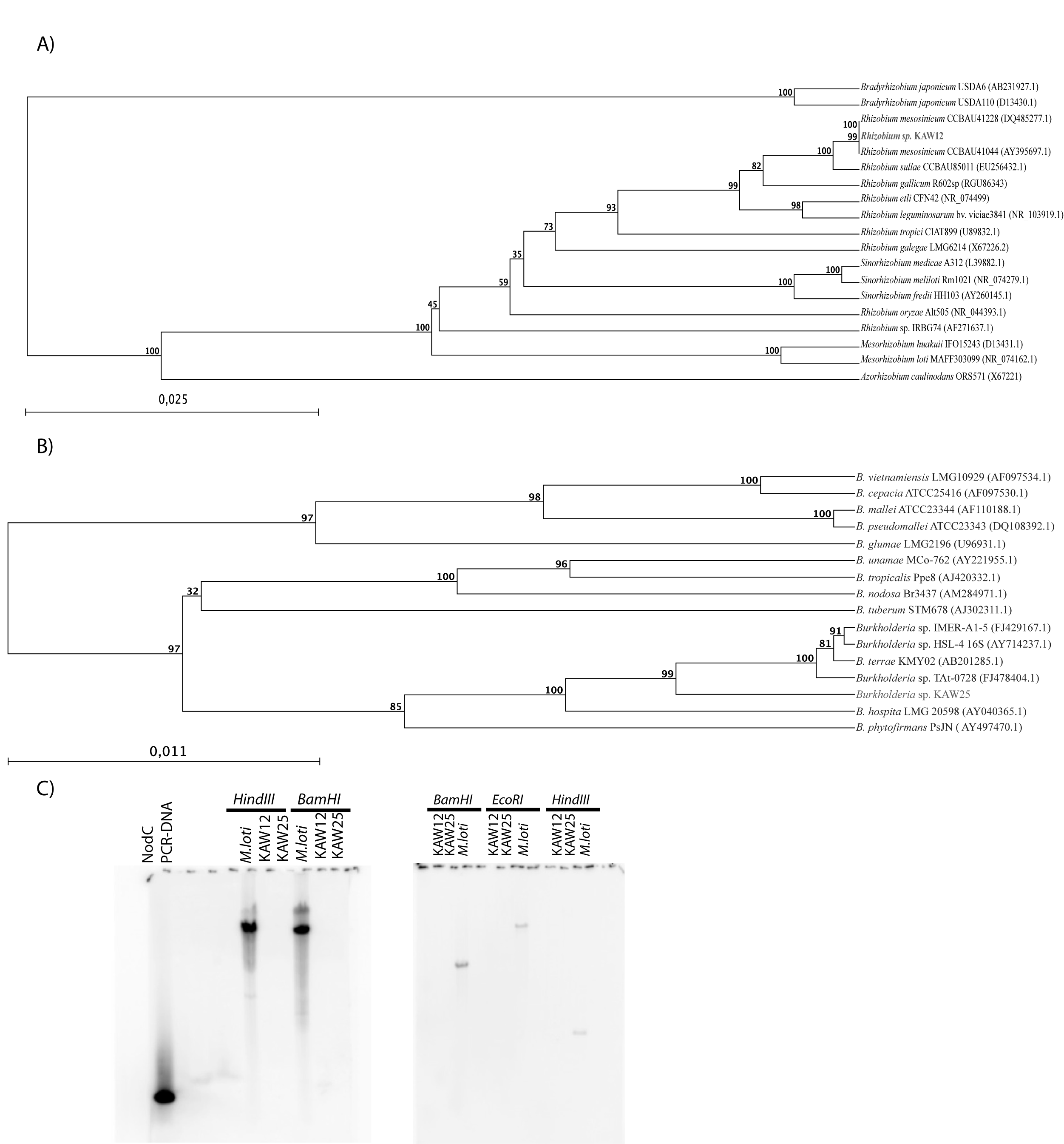

Supplement: S2 Fig — A) Phylogenetic relationship of KAW12 to other rhizobia strains based on 16S rRNA sequence. Bootstrap values are displayed on the tree nodes. (B) Phylogenetic relationship of KAW25 to other Burkholderia strains based on 16S rRNA sequence. Bootstrap values are displayed on the tree nodes. (C) Southern blot analysis illustrating the presence of NodC (left) and NifH (right) genes in the M.loti, but not in KAW12 and KAW25 bacteria. Bacterial DNA was digested with HindIII, BamHI or EcoRI restriction enzymes. (TIF) [file pgen.1005280.s002.tif]

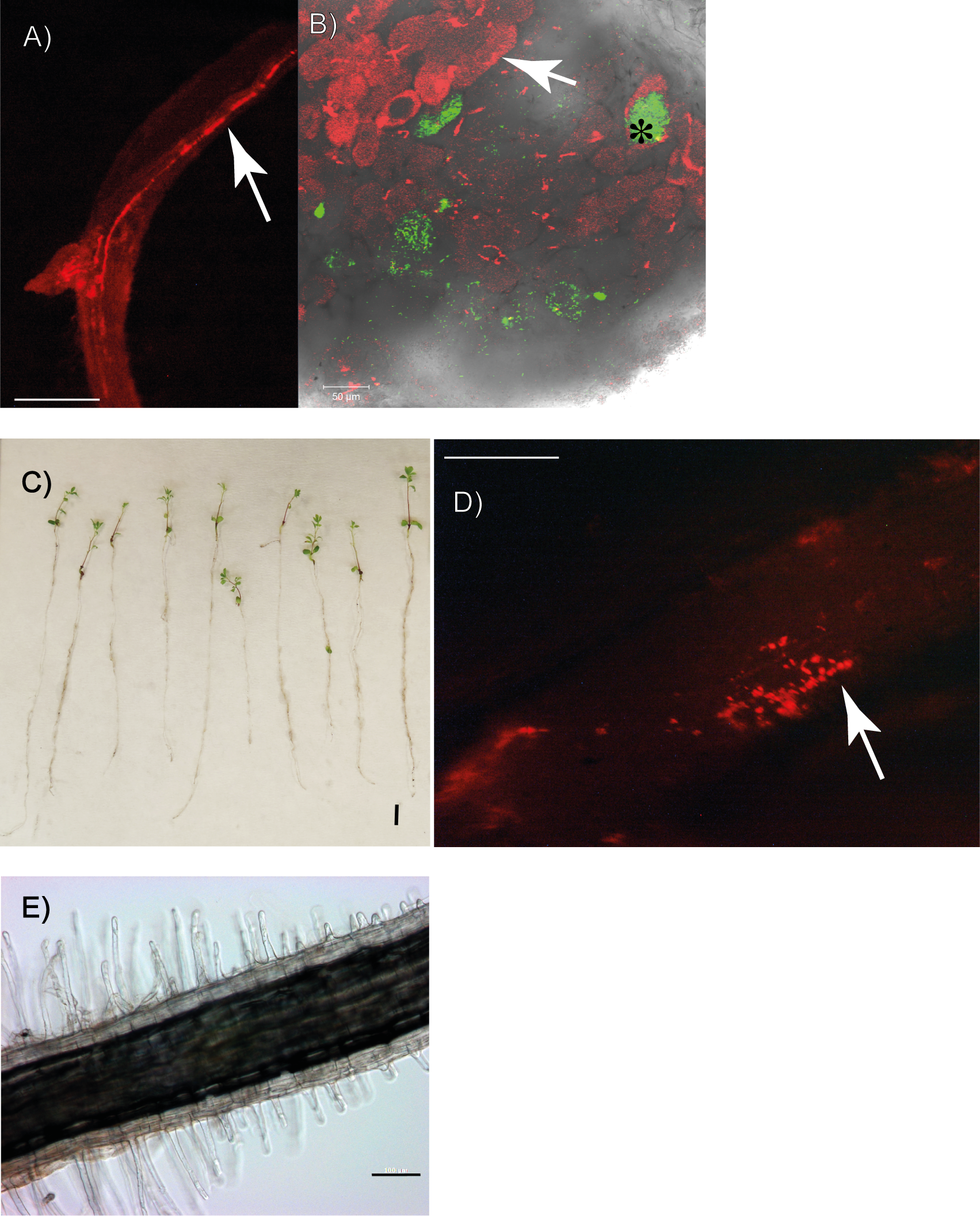

Supplement: S3 Fig — (A) KAW12 colonises Lotus roots endophytically. The arrow marks the presence of bacteria labelled with DsRED inside the root visualised with a fluorescent microscope (scale bar 500 μm). (B) Confocal image of a nodule section showing that KAW12 maintains its nodule colonisation capacity when is labelled with the GFP fluorescent protein (asterisk) and the nodule-inducing M. loti wild-type symbiont is labelled with the DsRED (arrow) (scale bar 50 μm). (C) KAW12DsRED isolated by antibiotic selection from infected nodules similar to the one presented in Fig 1B) induces a Nod minus, nitrogen starved phenotype (compare to Fig 1E) when applied to new Lotus plants (scale bar 1cm). (D) The isolated bacteria display DsRED fluorescence (arrow) when roots from (C) are visualised on fluorescence microscope (scale bar 200 μm). (E) KAW12 alone, or coinoculated with M.loti nodC (n = 45) was unable to induce root hair curling or microcolony formation (scale bar 100 μm). (TIF) [file pgen.1005280.s003.tif]

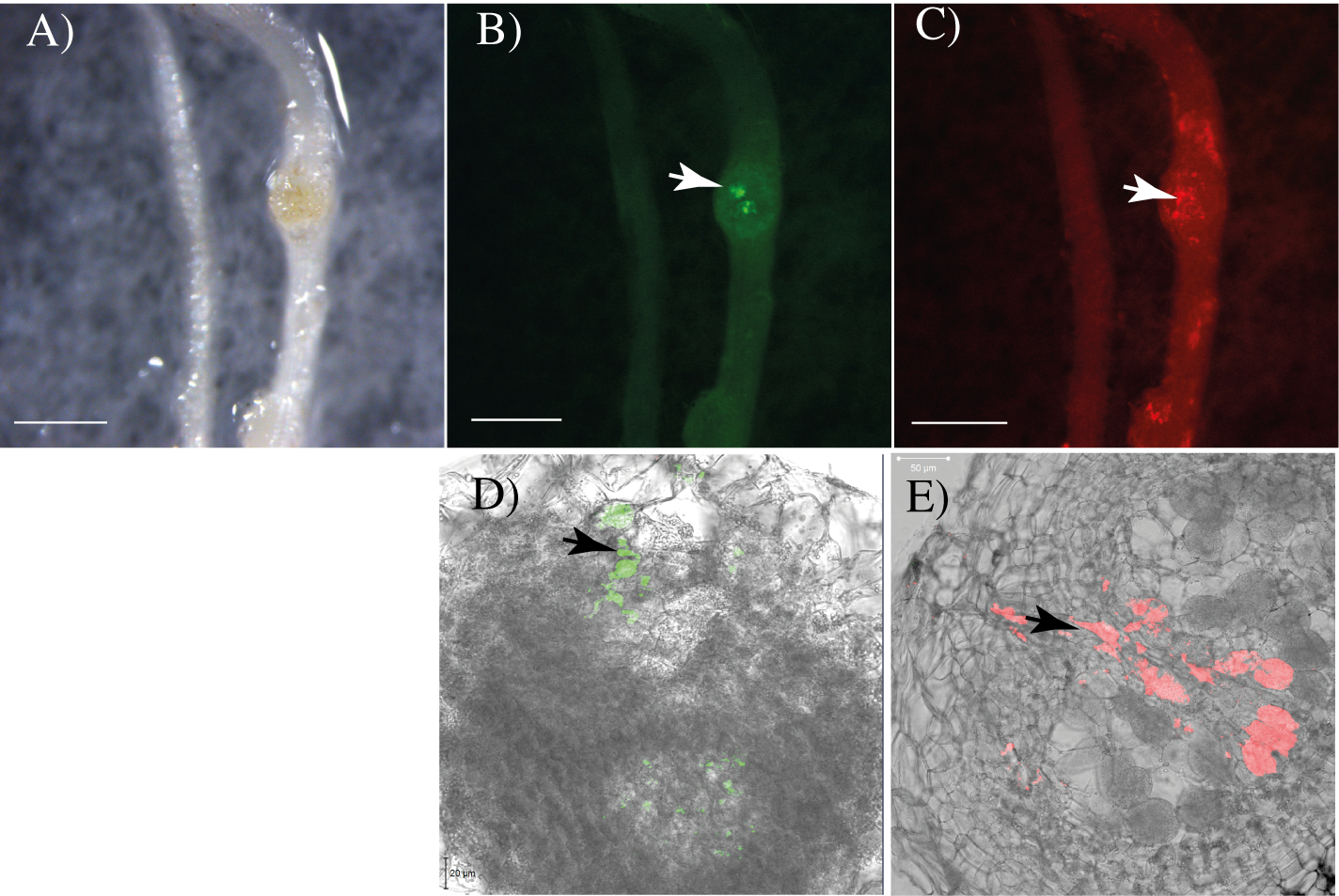

Supplement: S4 Fig — Whole nodule primordia visualised in bright field (A), with a GFP filter (B) or with a DsRED filter (C) showing the presence of A. caulinodans (arrow in B) and KAW12 (arrow in C) inside the primordia. Section of a nodule primordia induced by A. caulinodans and colonised by A. caulinodans (arrow in D) and KAW12 (arrow in E). A. caulinodanus GFP is visualised in green and KAW12DsRED in red. Scale bars in A to C- 500 μm. (TIF) [file pgen.1005280.s004.tif]

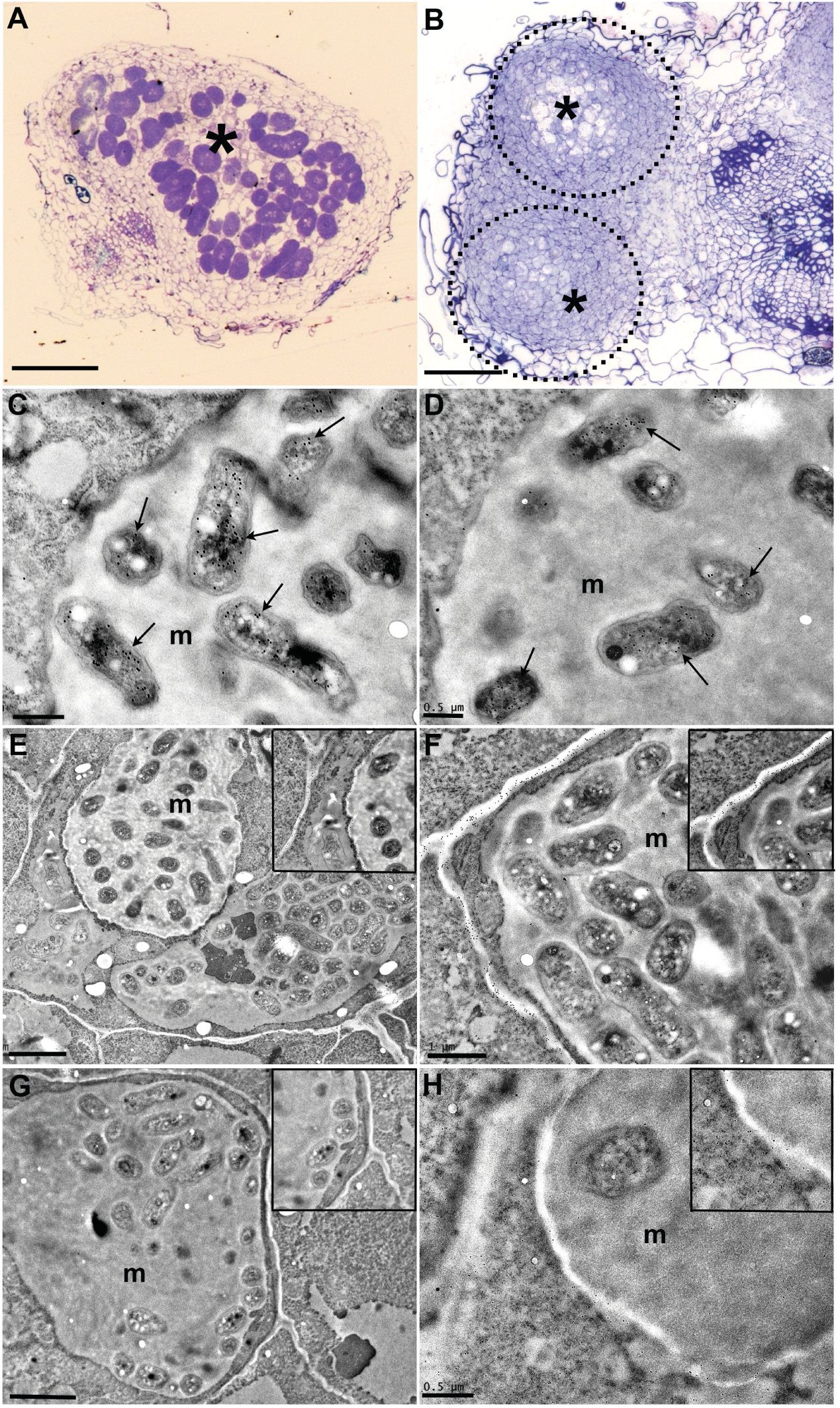

Supplement: S5 Fig — (A) Thin section of a mature nodule induced and colonised by M. loti exoUGFP displaying the infected cells (*) in the central zone. (B) Thin section of two closely developed nodule primordia (dashed lines) induced by M. loti exoU colonised by KAW12 (*). Compare with Fig 2H) to observe endophytic colonisation developing from the inner zone of the nodule. (C) and (D) Transmission electron micrographs of M. loti exoU induced nodules colonised by KAW12DsRED showing the immunogold labelling of KAW12 (arrows) using a DsRED antibody. (E) and (F). Transmission electron micrographs of M. loti exoU induced nodules colonised by KAW12DsRED showing the homogalacturonan epitope detection (arrows) using JIM5 antibody. (G) and (H). Transmission electron micrographs of M. loti exoU induced nodules colonised by KAW12DsRED showing the glycoprotein detection (arrows) using MAC236 antibody. Notice that KAW12 bacteria are surrounded by white undefined matrix (m) (C to H). Insets in (E) to (H) highlight the regions of interest. (TIF) [file pgen.1005280.s005.tif]

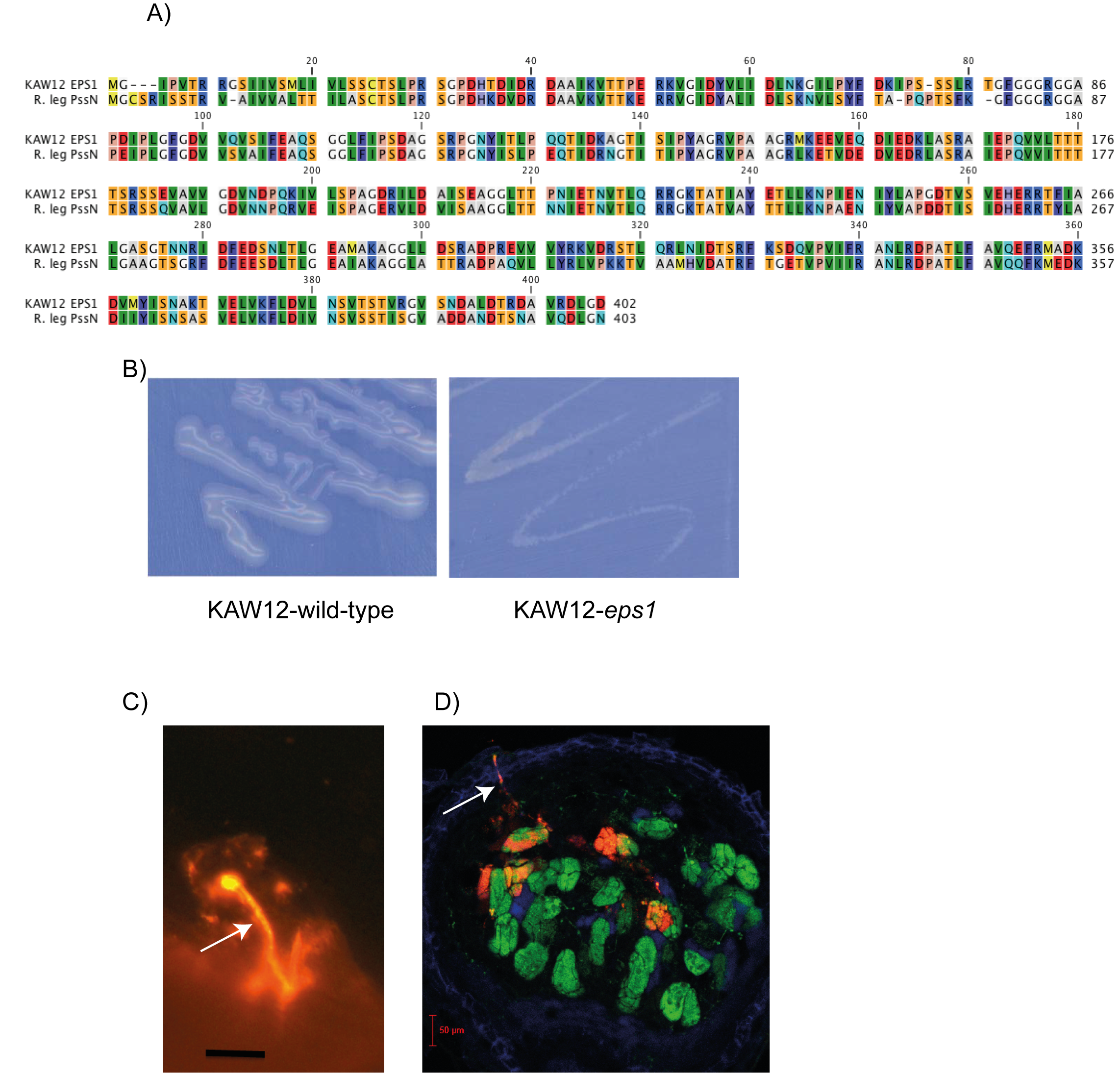

Supplement: S6 Fig — (A) Aminoacid alignment of KAW12 EPS1 predicted protein and the PssN protein from R. leguminosarum bv. trifolii. (B) The KAW12-eps1 mutant displays a non-mucoid phenotype when grown on plates. (C) The DsRED labelled KAW12-eps1 mutant colonises the root hair ITs (arrow) induced by M.loti exoU (scale bar 20μm). (D) The DsRED labelled KAW12-eps1 mutant colonises the root hair IT (arrow) and, with very low frequency, the nodules induced by M.loti wild type R7A_GFP. Nodule cell walls are visualised in blue using the DAPI filter on the confocal microscope (scale bar 50μm). (TIF) [file pgen.1005280.s006.tif]
