# Supplementary material for: A Legume Genetic Framework Controls Infection of Nodules by Symbiotic and Endophytic Bacteria
Source: PLoS Genet. 2015 Jun 4;11(6):e1005280. doi: 10.1371/journal.pgen.1005280 (PMC4456278; doi:10.1371/journal.pgen.1005280)
Supplement: S1 Text — (DOCX) [file pgen.1005280.s007.docx]

**S1 Text- Supporting Material and Methods**

**Plant material**

All plant mutants tested in this study originated from Gifu B-129 ecotype. A list of tested genotypes, and the primers used for genotyping is given in S1 Table.

**Bacterial strains**

All bacterial strains used in this work together with the antibiotics used for specific selection are given in S2 Table. The pFAJDsRED or pFAJGFP plasmids [[1](#_ENREF_1)] carrying the constitutively expressed DsRED or GFP were introduced into KAW12 by a triparental mating procedure to produce the KAW12DsRED or KAW12GFP, respectively. The pFAJGFP2 was introduced into *Burkholderia* KAW25 by triparental mating to produce the KAW25GFP strain. pFAJGFP2 contained *psbA* promoter driving the GFP gene transferred from pUTgfp2 vector [[2](#_ENREF_2)] in pFAJ1703 at the *Hpa*I site.

The primers used for bacterial DNA amplification are presented in S2 Table.

**Bacterial growth and inoculum preparation for plant inoculation**

All *M. loti* strains were cultured in YMB liquid medium supplemented with antibiotics at 28°C with constant agitation (200 rpm). *Rhizobium* sp. 129E and all strains of *Rhizobium* KAW12 were incubated in TY liquid medium with corresponding antibiotic selection at 28°C and shaking (200 rpm). *H. frisingense* GSF30 and *Burkholderia* KAW25 were grown in LB medium supplemented with antibiotics at 28°C and agitation (200 rpm), while *A. caulinodans* ORS571 – in antibiotic supplemented YEB liquid medium at 37 °C and 200 rpm mixing. Afterwards, the bacteria were diluted individually to OD_600_- 0.02. A 1/1 mixture of bacteria with the same OD_600_ was prepared prior to inoculation and was used for co-inoculation experiments.

**Plant growth**

Seeds were surface sterilized with 1% sodium hypochlorite for 20 min, germinated on sterile filter paper for 7 days, and transferred to containers (Magenta boxes in closed or open setup, or open plant trays [[3](#_ENREF_3)] containing clay granules supplemented with 1/4 B&D medium with 1mM KNO_3_ (60 ml for Magentas, 1500 ml for plant trays). After inoculation with bacteria, plants were incubated for 40 days at 21°C, 16/8h light/dark photoperiod, light intensity 200 μM, relative humidity 80%. Watering of the open set-up was done on a weekly basis using 1/4 B&D medium (50 ml per Magenta, and 500 ml per tray).

**Nodule colonisation analysis**

For nodule colonisation, the plants were removed from containers, the clay granules attached to the roots were removed by rinsing with water, and the plants were photographed. The whole root system was inspected using a Leica M165FC stereomicroscope. Each nodule was visualized using bright field, and with GFP and DsRED filters, and the bacterial infection in each nodule was recorded. For most of the experiments, the KAW12 DsRED and the *M. loti* GFP strains were used. KAW12 maintained its capacity for nodule colonisation also when the plasmids with fluorescence markers were swapped between the *M. loti* and the nonsymbiotic strains (S3B Fig). To verify that bacteria expressing DsRED inside the co-infected nodules is indeed KAW12, the bacteria were recovered from surface sterilized nodules; they were selected based on antibiotic resistance for the DsRED-containing plasmid, and were verified for their phenotype by inoculation on new *Lotus japonicus* wild-type plants (S3C, D Fig). The 16S rRNA sequence of five isolated colonies, combined with a nitrogen-starved, nodulation minus plant phenotype confirmed that the DsRED nodule-inhabiting bacteria were KAW12. In addition, nodules selected on the basis of internal fluorescence were surface sterilized and tested for the presence of the endophyte and/or symbiont by real-time amplification of DNA using 16S rRNA- or *NodC*-specific primers. The first primer set allows detection of both bacteria, while the second one detects the *M. loti* symbiont. Similar crossing point (CP) values were obtained when bacterial 16S rRNA was amplified, indicating the presence of similar amounts of bacterial DNA in the samples, but a large difference (more than 10 CP) was detected between KAW12- and the *M. loti* *exoU*-selected nodules when analysed for *NodC* gene amplification, indicating the preponderance of *exoU* bacteria in the GFP selected nodules (S3 Table).

**Confocal microscopy of root and nodule sections**

Single root pieces or nodules were embedded in 3.5% agarose, and sectioned (50μm) using a LeicaVT1000S vibratome. The sections were mounted on microscope slides and observed under the Zeiss LSM510 Meta and Zeiss LSM780 confocal microscopes using GFP (excitation wavelength: 488 nm, emission: 505-530 nm) and DsRED filters (excitation wavelength 543 nm, emission: 560-585 nm) at X20 and X40 magnification.

**TEM microscopy of nodule sections**

Nodules were fixed in 2.5 % glutaraldehyde in 0.1 M sodium cacodylate (pH 7.0) overnight at 4°C, dehydrated in an ethanol series, and embedded in LR White acrylic resin (Agar Scientific). Semithin sections (1 μm) were taken for light microscopy and ultrathin (70 nm) sections were taken for TEM using a Leica UCT ultramicrotome. The semithin sections were collected on glass slides and stained with 0.1 % toluidine blue, whereas the ultrathin sections were collected on pioloform-coated nickel grids and immunogold labelled with either monoclonal antibodies against MAC236 and JIM5 raised in rats (gifted by N.J. Brewin), or with polyclonal antibodies against DsRed and GFP raised in rabbits (Abcam). The sections were incubated for 2 h in the various primary antibodies (diluted 1:10), followed by 30 min incubation in an anti-rat secondary antibody raised in rabbit (diluted 1:400) (Southern Biotechnology) for the MAC236 and JIM5 antibodies, and then for 1 h in 15 nm goat anti-rabbit gold (GE Healthcare) (diluted 1:100) (for all the antibodies). The ultrathin sections were then stained with uranyl acetate for 10 min before being viewed and digitally photographed using a JEM 1400 transmission electron microscope (JEOL).

**Southern blot analysis**

For Southern blot analyses, 10 μl of bacterial genomic DNA was digested with appropriate restriction enzymes, followed by long (8 h) agarose gel (0.7 %) electrophoresis at low voltage (40 V). The gel was depurinated (0.25 M HCl) for 15 min, denatured (1.5 M NaOH, 1.5M NaCl) for 30 min, and neutralized (0.5 M Tris-HCl pH 7.4, 3 M NaCl) twice for 15 min. A capillary transfer was assembled and left for DNA transfer for 8 h. The DNA transfer from the gel to the membrane was determined by UV light exposure of the gel. The membrane was washed in 10xSSC buffer and cross-linked using Stratalinker (Stratagene). A radioactive labelled probe was synthesized via PCR reaction, using the *NodC* and *NifH* primers (S2 Table). The hybridization was performed for 8 h at 65°C, and the final wash was made in 1x SSC, 0.1% SDS. Storage phosphor screens (Molecular Dynamics) were exposed to membranes for 48h, followed by a scan with Typhoon TRIO Variable Mode Imager (Amersham Biosciences).

**References**

1. Kelly SJ, Muszynski A, Kawaharada Y, Hubber AM, Sullivan JT, Sandal N, et al. Conditional requirement for exopolysaccharide in the Mesorhizobium-Lotus symbiosis. Molecular plant-microbe interactions : MPMI. 2013 Mar;26(3):319-29.

2. Errampalli D, Leung K, Cassidy MB, Kostrzynska M, Blears M, Lee H, et al. Applications of the green fluorescent protein as a molecular marker in environmental microorganisms. J Microbiol Meth. 1999 Apr;35(3):187-99.

3. Tirichine L, James EK, Sandal N, Stougaard J. Spontaneous root-nodule formation in the model legume Lotus japonicus: a novel class of mutants nodulates in the absence of rhizobia. Molecular plant-microbe interactions : MPMI. 2006 Apr;19(4):373-82.
