# Supplementary material for: A Legume Genetic Framework Controls Infection of Nodules by Symbiotic and Endophytic Bacteria
Source: PLoS Genet. 2015 Jun 4;11(6):e1005280. doi: 10.1371/journal.pgen.1005280 (PMC4456278; doi:10.1371/journal.pgen.1005280)
Supplement: S1 Table — (DOCX) [file pgen.1005280.s008.docx]

**Supporting Table 1**

**Plant genotypes and primers used for genotyping *Lotus* mutants**

| **Genotype** | **Primer sequence** |
| --- | --- |
| arpC1(2-3) | 5’ CTCTTCCCCACCAGTCCACCAC 3’  5’ ACACCATTAGTGATTGAGCACC 3’ |
| npl1-1 | 5’ TCACAGGAGCATCATTGAGCATAGG 3’  5’ GTATATTGAACAACTACCACCTAC C3’ |
| cerberus (sym7) | 5’ ACATCATCTCCAGACATTAGC 3’  5’ GTGGTGATTACCTGCCATTTAG 3’ |
| sst1 (sym13) | 5’ TTATAGATTAGTCACTGTTGTCC 3’  5’ GCATGGATTATGGTCTCATGATC 3’ |
| nap1(sym67) | 5’ CATGGCAGTCTTAGGAAGCTCTAC3’  5’ TTGGTCAATACTCGACAATTCATAG 3’ |
| cyclops2 | 5’ CTCATGTTCATCTAACTTCAACAC 3’  5’ CTTCACCAATAGAGTCTACTTGAAC 3’ |
