# Supplementary material for: A Legume Genetic Framework Controls Infection of Nodules by Symbiotic and Endophytic Bacteria
Source: PLoS Genet. 2015 Jun 4;11(6):e1005280. doi: 10.1371/journal.pgen.1005280 (PMC4456278; doi:10.1371/journal.pgen.1005280)
Supplement: S2 Table — (DOCX) [file pgen.1005280.s009.docx]

**Supporting Table 2**

**A. Bacterial strains used in this study**

|  | \| Strain \| Antibiotic selection [μg/ml] \| Reference \| \| --- \| --- \| --- \| \| *Herbaspirillum frisingense* GSF30 GFP \| Kanamycin50 \| Kindly provided by A. Hartmann \| \| *Herbaspirillum* B501 GFP \| Kanamycin100 \| Kindly provided by A. Hartmann \| \| *Rhizobium giardinii* 129E RFP \| Streptomycin200Gentamycin15 \| Kindly provided by M. Roth and  P. Schulze-Lefert \| \| *Burkholderia* KAW25 \| Tetracyclin5 \| Kindly provided by K. Minamisawa \| \| *Burkholderia* KAW25 GFP \| Tetracyclin5 \| This work \| \| *R. mesosinicum KAW12* \| Phosphomycin_50_ \| Kindly provided by K. Minamisawa \| \| *R. mesosinicum* KAW12 DsRED \| Tetracyclin_10_, Phosphomycin_50_ \| This work \| \| *R.mesosinicum* KAW12 GFP \| Tetracyclin_10_, Phosphomycin_50_ \| This work \| \| *R. mesosinicum KAW12 eps1* \| Neomycin_100_ \| This work \| \| *R. mesosinicum* KAW12 *eps1* DsRED \| Tetracyclin_10_, Neomycin_100_ \| This work \| \| *Azorhizobium caulinodans* ORS571-GFP \| Carbamycin_100_, Gentamycin_25_ \| Kindly provided by S. Goormachtig \| \| *Mesorhizobium loti* R7A GFP \| Tetracyclin_2_ \| Kindly provided by J. Sullivan \| \| *Mesorhizobium loti* MAFF 303099 DsRED \| Phosphomycin_100_, Gentamycin_10_ \| Maekawa et al, 2010 \| \| *Mesorhizobium loti* R7A *exoU* GFP \| Tetracyclin_2_, Neomycin_50_ \| Kelly et al, 2013 \| \| *Mesorhizobium loti* R7A *nodZ* GFP \| Kanamycin_20_ \| Rodpothong et al, 2009 \| |
| --- | --- | --- | --- | --- | --- | --- | --- | --- | --- | --- | --- | --- | --- | --- | --- | --- | --- | --- | --- | --- | --- | --- | --- | --- | --- | --- | --- | --- | --- | --- | --- | --- | --- | --- | --- | --- | --- | --- | --- | --- | --- | --- | --- | --- | --- | --- | --- | --- | --- |

**B. Primers used for specific bacterial DNA amplification**

| Name | Primer sequence |
| --- | --- |
| *M.loti* *NodC* | 5’ TACTGTTGCCATCTGCTCTT 3’  5’ ACCGCTTGATCTTGCATCTT 3’ |
| *M.loti* *NifH* | 5’ ACGGCGCCTATGATAATGTC 3’  5’ ATTTGCTCGTCCGTCTTCAT 3’ |
| 16S rRNA | 5’ AGAGTTTGATCCTGGCTCAG 3’  5’ AAGGAGGTGATCCAGCC 3’ |
| DsRED | 5’ GGACGTCATCAAGGAGTTCA 3’  5’ AAGTTCATCACGCGCTCC 3’ |
| GFP | 5’ TTTTCACTGGAGTTGTCCCA 3’  5’ TGCTAGTTGAACGCTTCCAT 3’ |
