# Supplementary material for: A Legume Genetic Framework Controls Infection of Nodules by Symbiotic and Endophytic Bacteria
Source: PLoS Genet. 2015 Jun 4;11(6):e1005280. doi: 10.1371/journal.pgen.1005280 (PMC4456278; doi:10.1371/journal.pgen.1005280)
Supplement: S3 Table — (DOCX) [file pgen.1005280.s010.docx]

**Supporting Table 3**

**Real time PCR CP values for 16S rDNA and *NodC* on nodule primordia selected based on microscopy fluorescence**

| Type of primordia | 16S rDNA | *NodC* |
| --- | --- | --- |
| uninfected | 33,28 | 37,67 |
| *M.loti exoU*_GFP selected | 27,52 | 23,07 |
| KAW12_DsRED selected | 29,76 | 33,19 |
| *M.loti exoU*+KAW12_GFP+dsRED selected | 27,97 | 25,21 |
